# 3D-Printed Cold Preservation Device in Renal Autotransplantation for the Treatment of a Patient With Renal Artery Stenosis

**DOI:** 10.3389/fbioe.2021.738434

**Published:** 2022-01-03

**Authors:** Dong Cui, Bin Wu, Dali He, Yanen Wang, Yong Jiao, Bo Zhang

**Affiliations:** ^1^ Department of Urology, Tangdu Hospital, The Air Force Medical University, Xi’an, China; ^2^ Industry Engineering Department, School of Mechanical Engineering, Northwestern Polytechnical University, Xi’an, China

**Keywords:** 3D print, polylactide, kidney diseases, kidney transplantation, laparoscopic surgery, rat

## Abstract

Percutaneous transluminal angioplasty (PTRA) is a common treatment method for renal vascular disease (RVD). However, PTRA may not be effective in patients with abnormal vascular disease. Renal autotransplantation (RAT) has been used as an alternative therapy for these diseases. Restrictions due to intracorporeal kidney cold preservation and the renal function of intracorporeal RAT were not as well protected compared with open operation. We developed this technique of 3D-printed polylactide (PLA) cold jackets for laparoscopic complete intracorporeal RAT for the purpose of better protecting the renal function and determining the feasibility of this novel procedure. The procedure was successfully applied to a 51-year-old woman with bilateral renal artery stenosis. The operation time was 5 hours, and blood loss was 200 ml. The patient’s blood pressure remained constant throughout the operation, and the pressure was maintained at 120-140/70–90 mmHg without antihypertensive drugs 1 week after the operation. B-ultrasound showed that the blood flow signal of the transplanted kidney was normal and the boundary between the skin and medulla was clear. The patient was discharged 2 weeks after surgery. One year postoperatively, Doppler ultrasound of the autotransplant showed that the transplanted kidney was normal in size and shape. Radionuclide renal dynamic imaging revealed that the glomerular filtration rate (GFR) of the transplanted kidney was 36.9 ml/min. 3D-printed polylactide (PLA) cold jackets for laparoscopic complete intracorporeal RAT are a safe and effective method for the treatment of renal artery stenosis and represent a feasible method for preserving the renal function of severe renal artery stenosis patients; however, the technology is still at the exploratory stage and has room for further improvements.

## Introduction

3D printing technology is a new manufacturing process that gradually developed after the 1980s. 3D printing (rapid prototyping or additive manufacturing) is the process of creating solid 3D objects from a collection of images in the form of a digital file. The printer deposits layers of materials (such as plastic, resin, or metal) in a volumetric manner such that a replica of the object is obtained. This innovative technology will likely revolutionize our knowledge and understanding of the structure of human tissues and organs, and it also has therapeutic applications. In recent years, as biomedical polymer materials have been widely used in the field of medicine, the use of 3D printing technology to prepare biomedical polymer materials has attracted the attention of many researchers and achieved important results, such as bone repair projects in orthopedics and neurology, nerve tissue engineering scaffold material projects, and surgical planning projects. These projects have produced ideal repair effects and greatly reduced medical costs.

Renal vascular disease (RVD) is a rare but important cause of hypertension. Because essential hypertension is increasingly common and blood pressure is not commonly measured, RVD is not usually diagnosed at an early stage. Percutaneous transluminal angioplasty (PTRA) is a common treatment method for RVD (Buck et al., 2016), and this technology has improved over time and is now increasingly used, such as for placing vascular stents at the level of artery stenosis after forced dilation in a balloon (Kari et al., 2015; Chavent et al., 2017; Agrawal et al., 2018; Bacic et al., 2020; Raborn et al., 2020). This method may provide a minimally invasive alternative for certain patients who appear to be resistant to PTRA (Kim et al., 2017; Safdar et al., 2020).

However, PTRA may not be effective and could even be dangerous in patients with dysplasia or atherosclerotic lesions involving infrahilar branches or inflammatory lesions, such as those associated with Takayasu disease (TD). Therefore, the role of PTRA in the treatment of renovascular hypertension (RVH) is still controversial, especially in patients who present complicated renal vascular lesions or patients for whom antihypertensive drugs either are ineffective or carry a potential risk of acute renal failure.

At present, renal autotransplantation (RAT) has been used as an alternative therapy for renovascular hypertension. Traditional renal autotransplantation using an open technique with one or two incisions is required for nephrectomy or anastomosis of renal vessels. Currently, the benefits of laparoscopic surgery are well established and have led to its widespread adoption, including in the field of transplantation (Giacomoni et al., 2016; Cantrell and Oberholzer, 2018; Choi et al., 2018; Perkins et al., 2018; Spinoit et al., 2019). Some studies reported the use of laparoscopic and robot renal autotransplantation (RAT). In most studies, the use of laparoscopy is limited and mostly applied for nephrectomy (Hiess and Seitz, 2016; Siena et al., 2019; Kishore et al., 2020). After nephrectomy, renal vessels are extracted from the abdominal cavity and transferred to the bench table, and a separate incision is required to anastomose the renal vessels with the iliac vessels (Kubota et al., 2020; Pomy et al., 2020; Smith et al., 2020). Although some studies have reported intracorporeal robot-assisted renal autotransplantation and restrictions due to kidney cold preservation *in vivo*, the kidney is not cold preserved continuously during the operation after vessel dissociation (Breda et al., 2021). The results of these studies suggest that compared with intracorporeal renal autotransplantation, faster renal function recovery and shorter cold ischemia time occur with extracorporeal robot-assisted renal autotransplantation, suggesting that in intracorporeal renal autotransplantation, renal function was not best protected. At present, the use of continuous cold preservation in laparoscopic complete intracorporeal of human renal autotransplantation has not been reported.

Hypothermic perfusion and preservation technology are necessary conditions for renal autotransplantation. Cold perfusion can provide sufficient time and an appropriate operating environment for autologous kidney transplantation. Hypothermic perfusion fluid can protect renal function during renal ischemia (Lin et al., 2020), but one disadvantage of this method of continuous perfusion is that the effect is not accurate, and cell necrosis, apoptosis, and acute tubular necrosis are caused by blood supply recovery, especially when blood vessel anastomosis cannot be carried out (Urbanellis et al., 2020). The common method is adding ice water during suture, but continuous insertion of ice slush in the abdominal cavity not only distracts the surgeon and increases operative time but also is a rudimental method that does not guarantee a homogeneous parenchymal cooling effect with the possibility of compromising graft function. Cold ischemia preservation is the best preservation method (Kaths et al., 2017). A device covering the whole renal surface isolates the kidneys from surrounding tissue and maintains a constant low temperature, potentially preserving the renal function for a longer time, and by an insulation layer, it avoids cooling-related complications to the surrounding abdominal organs; this may be a perfect way to preserve the kidney. 3D solid models according to the shape of the kidney, and the greatest advantage of 3D printing is that artificial implants can be customized accurately and effectively. So we use 3D printing technology to develop a cooling jacket that consists of two sealed films that completely cover the kidney to form a channel for the cooling solution from one end to the other, and apply cold ischemia preservation continuously for the kidney.

This 3D-printed cold jacket can completely cover the graft, avoid the gradual melting of ice in the abdominal cavity, control the kidney temperature, and reduce the potential graft impairment, and it can be continued during the process of suturing. Its effect may be equivalent to that of cold preservation *in vitro*. Currently, some studies reported the use of cold preservation devices for pig intraperitoneal kidney transplantation (Menon et al., 2014; Meier et al., 2018; Samuels et al., 2019). However, they are all in the exploratory stage. There were no reports on the use of this 3D printing device in completely minimally invasive human kidney transplantation.

Polylactide (PLA), a kind of linear thermoplastic aliphatic polyester, is mainly prepared from starch raw materials through saccharification, fermentation, and certain chemical reactions. PLA has good biocompatibility and biodegradability and can be completely degraded under specific conditions, and the final products are carbon dioxide and water (Madhavan Nampoothiri et al., 2010; Tyler et al., 2016). In addition, PLA also has good thermal stability, solvent resistance, excellent gloss, transparency, resistance to certain bacteria, and flame retardancy (Farah et al., 2016). Based on the advantage of its unique plasticity and transparency, 3D-printed polylactide (PLA) cold jackets can be used completely intracorporeally. We can clearly observe the kidney condition and the color of the kidney during the whole process of vascular anastomosis, and it is convenient for operation and can apply cold ischemia preservation continuously during the operation after vessel dissociation; thus, the kidney function can be protected to the maximum extent. This technology may make complete intracorporeal renal autotransplantation convenient, effective, and safe. Such surgery may be unique with no incision to extract or introduce kidneys, and cold ischemia preservation can be applied as same as the *in vitro* approach. Currently, there have been no reports on the use of this continuous cold ischemia preservation technology in laparoscopic complete intracorporeal of human renal autotransplantation. We developed this 3D-printed polylactide (PLA) cold jacket technique for completely intracorporeal laparoscopic RAT for the purpose to investigate the feasibility of this novel procedure and describe the first successful application of this concept in the treatment of renal artery stenosis.

## Materials and Methods

### Patient

**FIGURE 1:**
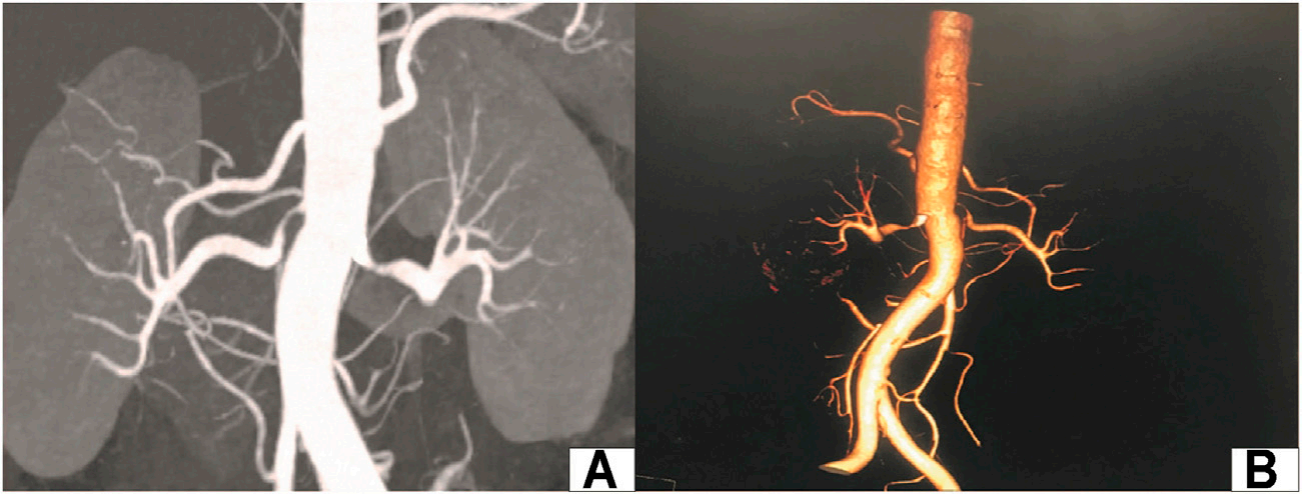
Preoperative images of the renal artery. **(A)**: CT renal angiography; **(B)**: CT 3D reconstruction of renal vessels.

The patient is a 51-year-old woman who was admitted to the urology department of our hospital on February 21, 2017. The blood pressure of the patient was elevated and fluctuated in the range of 160-200/90–100 mmHg. Nifedipine and indapamide were used, which provided poor blood pressure control. Thyroid functions and blood biochemical examination showed no extensive abnormalities. The plasma renin activity, angiotensin, and aldosterone were normal. Ultrasonic examination showed that the inner diameter at the beginning of the left and right renal arteries was 2.2 and 1.5 mm, respectively. Renal artery CTA showed 80% stenosis in the proximal segment of the left renal artery and 90% stenosis in the initial segment of the right renal artery **(** Figures 1A,B **)**. The CT examination of the adrenal glands showed no significant abnormalities in either adrenal glands. Bilateral radionuclide renal dynamic imaging showed that the glomerular filtration rate (GFR) of the left and right kidneys was 56.0 ml/min and 21.5 ml/min, respectively. Complete intracorporeal laparoscopic right kidney renal autotransplantation was proposed. After the operation, the operation time, bleeding loss, hot ischemia time (from ligation of the right renal artery to initiation of renal cold perfusion), cold ischemia time (from initiation of renal cold perfusion and preservation to completion of venous anastomosis), time of venous and arterial anastomosis, postoperative blood pressures, and GFR were recorded.

### 3D Printing

Thin-layer CT scan images of the patients’ kidneys were extracted, DICOM format files were extracted from the CT scans, and images were processed with Mimics 17.0 software (Materialise Inc., Leuven, Belgium). After using threshold selection, region growth, multilayer editing, and modification techniques, three-dimensional reconstruction images of different parts of the kidney were obtained and then combined into a complete kidney. Using Mimics 17.0 software (Materialise Inc., Leuven, Belgium) cold jackets were designed.

The cool jacket is 2 mm larger than the real kidney, which is convenient for operation. The cooling jacket consists of two sealed films that completely cover the kidney to form a channel for the cooling solution from one end to the other. The equipment connects inflow and outflow to form a circuit, and the cooling solution operates at a constant volume and temperature, isolating the kidneys from surrounding tissue, which avoids the injury of adjacent abdominal organs and the heat and excessive cold injury of the kidney. The transparent and elastic PLA material is used for 3D rinting, suitably maintains the temperature of the kidney, and allows easy extraction of the device. A window was designed to access the renal hilum for vascular anastomosis, while the kidney is still in the cold jacket. Finally, a standardized STL file of the cool jacket for 3D printing was output.

**FIGURE 2:**
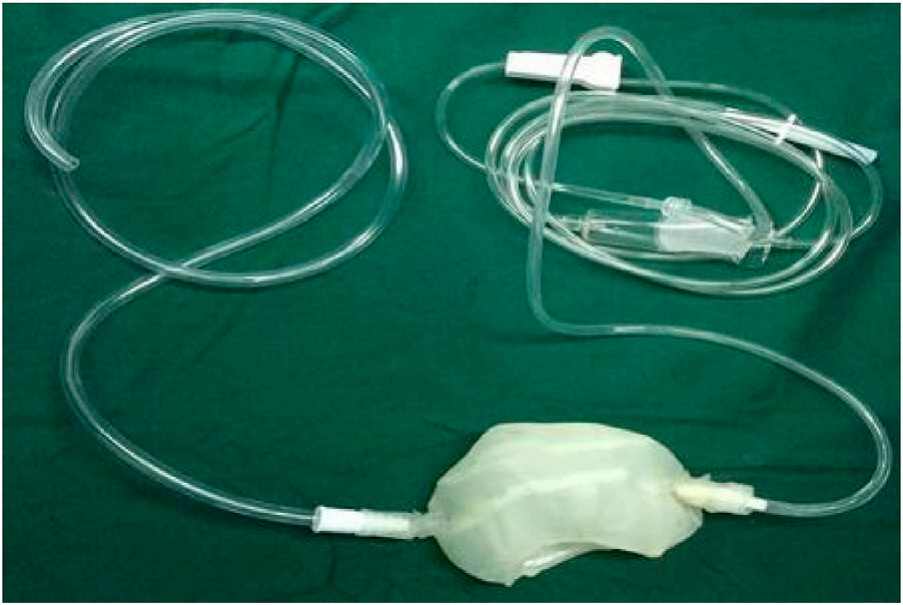
3D printing cold jacket of the kidney.

The STL files were formatted to meet printing parameters of the 3D printer (Shanghai liantai Technology Co., Ltd. rs4500 China). A PLA filament (Nature Work Inc., USA) is extruded from a 0.3-mm nozzle at the optimum temperature of 210°C. The external thickness of the cool jacket was 1.5 mm, the internal thickness was 1 mm, the distance between the two layers is 2 mm, and the diameter of inflow and outflow pipes is 3 mm. The printing time is 8 hours; after the printing, the cold jacket is rinsed in 70% ethanol overnight and sterilized for 90 min using the low-temperature plasma hydrogen peroxide sterilizer (Shandong Xinhua Medical Instrument Co., LTD., SQ-D, China) (Figure 2).

### Surgical Technique

Complete intracorporeal laparoscopic right renal autotransplantation was performed. General anesthesia and indwelling catheter: a 10-mm trocar was inserted at the outer edge of the rectus abdominis parallel to the umbilicus as the laparoscopic lens hole. Approximately 6 cm from the laparoscopic lens hole, two 10-mm trocars were inserted at the hypogastrium and medial side of the anterior superior iliac spine (as an isosceles triangle). The right external iliac artery and vein, proximal to the bifurcation of the iliac vessels, distal to the abdominal wall, were carefully dissociated. Approximately 4 cm from the umbilicus at the edge of the rectus abdominis, a 10-mm trocar was inserted as the laparoscopic lens hole, and approximately 3 cm from the costal margin, a 10-mm trocar was inserted in the midclavicular line.

After the colon was dissociated, the renal vessels were completely dissociated to the maximum extent and completely dissociated from the right kidney. The excess perirenal fat was removed, and the fat that maintained the blood supply of the ureter between the lower pole of the kidney and the ureter was retained, and the adipose tissue around the ureter was properly retained. The ureter was distally dissociated as far as possible, and the upper and lower poles of the right kidney were bound with yarn strips as the extraction points of the kidney. From the root of the renal arteriovenous artery, the renal artery was ligated using laparoscopic clips close to the aorta, and the renal vein was then ligated in a similar manner. The renal artery and vein were then transected above the clips, and arteriovenous dissociation was carefully performed.

**FIGURE 3:**
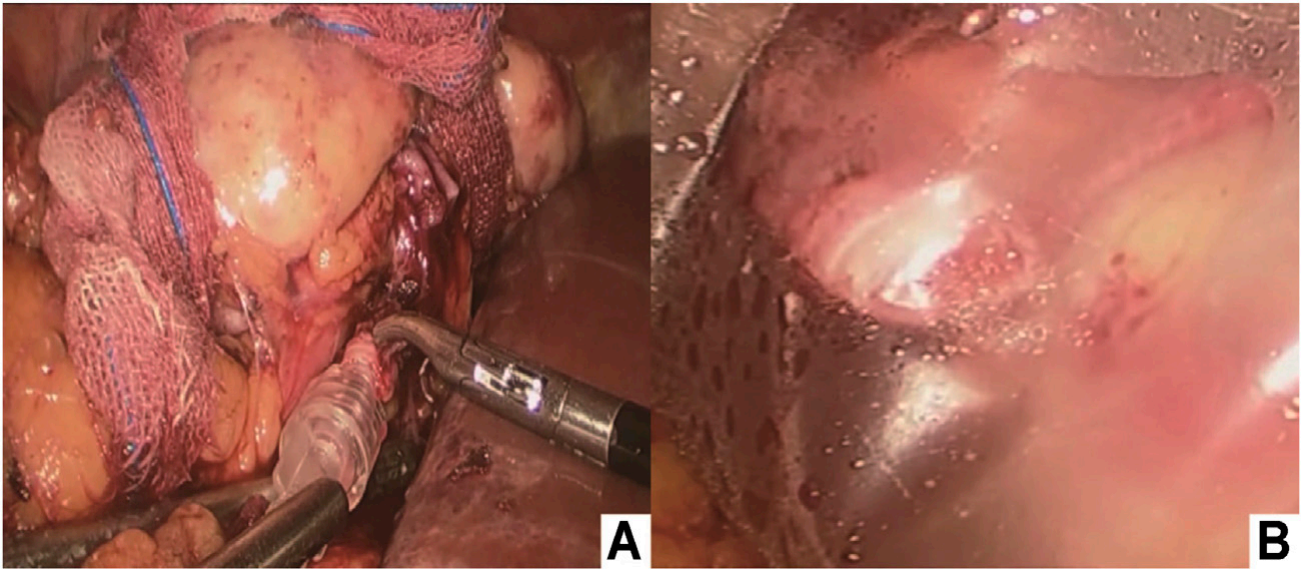
**(A)** Intracorporeal cold perfusion of the kidney using a cannula and tubing passed through the assistant port to allow renal artery perfusion until clear effluent was seen from the renal vein and the color of the right kidney was pale; **(B)** 3D-printed cold jacket was completely covered on the surface of the free kidney.

**FIGURE 4:**
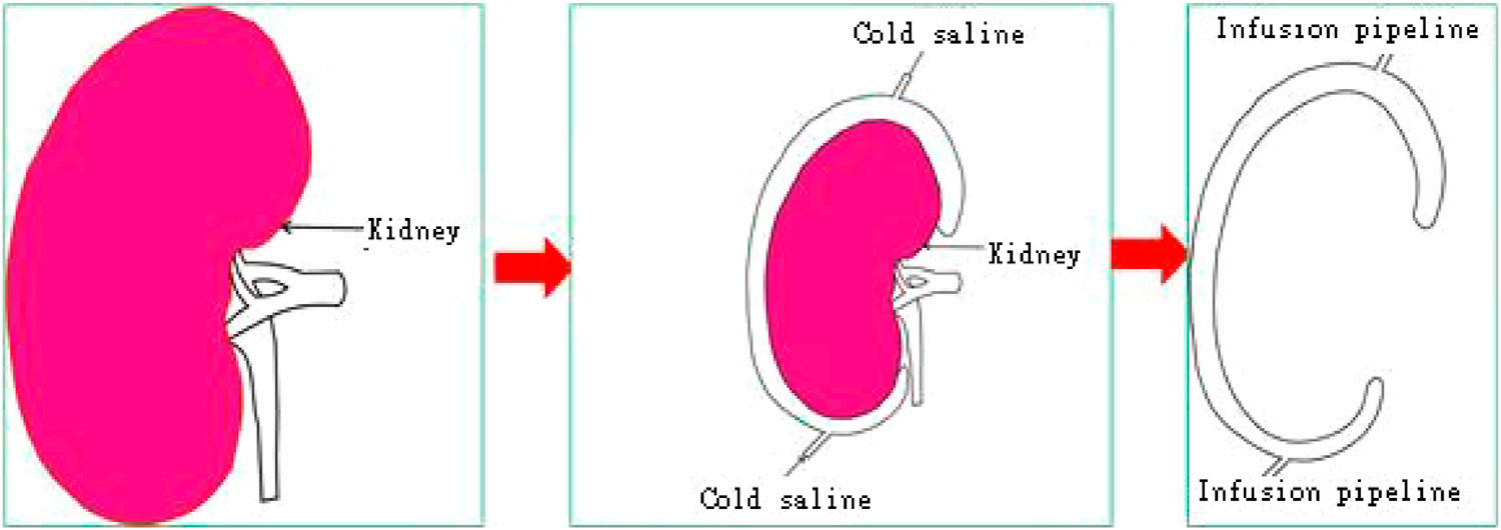
3D-printed model of the cold jacket covered on the surface of free kidney.

Immediately after dividing the vessels, the perfusion cannula was inserted in the transected artery lumen, which was continuously flushed with ice-cold perfusion liquid (hypertonic purine citrate fluid) solution under gravity until the clear effluent was seen from the renal vein and the color of the right kidney was pale (Figure 3A). The 3D-printed cold jacket completely covered the surface of the free kidney, and ice-cold saline water was continuously infused through the infusion pipeline. The kidney was carefully placed in the pelvis, and the opening of the renal artery and vein were placed close to the external iliac vessels (Figure 3B; Figure 4).

The external iliac vein was clamped with laparoscopic bulldog clamps, and a venotomy incision was made. A running end-to-side anastomosis was created between the renal vein and the external iliac vein using a 5-0 Prolene line. Before the last suture was placed, the lumen was irrigated with heparinized saline through a 5-Fr ureteral catheter to remove intraluminal air. After completing the venous anastomosis, a bulldog clamp was placed on the renal vein, and the clamps were released from the external iliac vein. End-to-side arterial anastomosis was performed, similar to that for the vein. The 3D-printed cold jacket was then removed from the surface of the kidney. Upon completion, the clamps were removed, beginning with the distal external iliac artery, followed by the renal vein and then the proximal external iliac artery.

## Results

**FIGURE 5:**
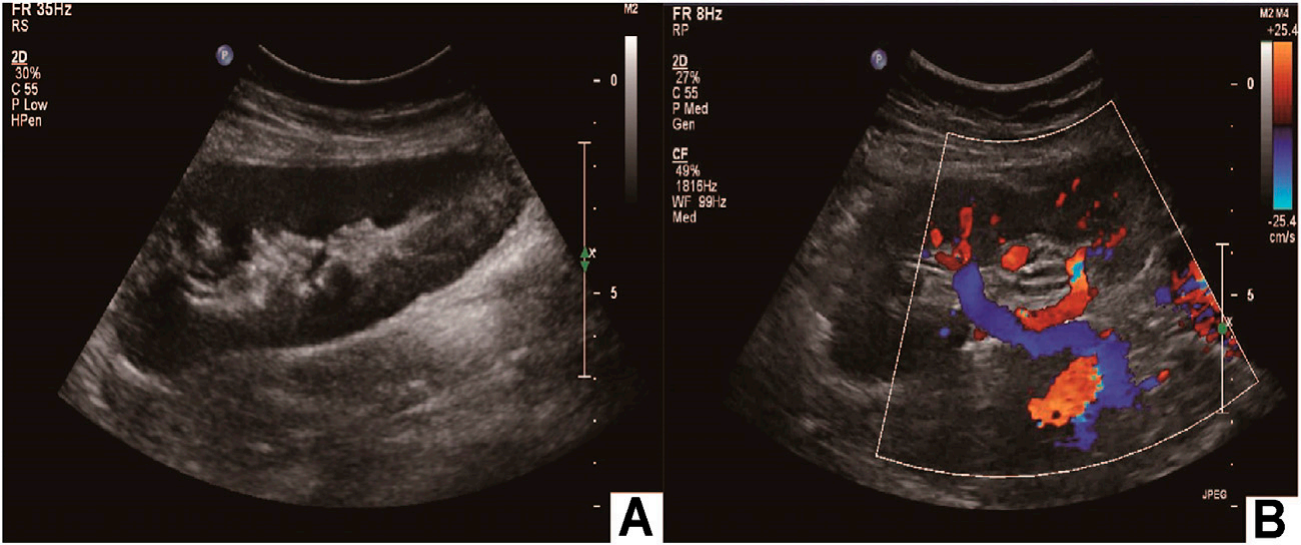
Postoperative imaging confirmed autotransplantation success. **(A)** Doppler ultrasound of autotransplant showing that the transplanted kidney was of normal size and shape; **(B)** the transplanted kidney was supplied by the right external iliac artery, and the vein returned to the right external iliac vein.

The operation time was 5 hours, and the hot ischemia time (from ligation of the right renal artery to initiation of renal cold perfusion) was 2 min. The cold ischemia time (from initiation of renal cold perfusion and preservation to the completion of venous anastomosis) was 76 min. The time for venous and arterial anastomosis was 23 and 27 min, respectively. In addition, the estimated blood loss was 200 ml (Table 1). The blood pressure remained constant throughout the operation. Postoperative rehydration and infection prevention were administered. Blood pressure monitoring was maintained at 120-140/70–90 mmHg without antihypertensive drugs. The patient was discharged 2 weeks after surgery. One 1 year postoperatively, Doppler ultrasound of the autotransplant showed that the transplanted kidney was normal in size and shape, and the boundary between the skin and medulla was clear (Figure 5A). The blood flow signal of the transplanted kidney was not abnormal, the transplanted kidney was supplied by the right external iliac artery, and the vein returned to the right external iliac vein (Figure 5B). The arteriovenous phase and excretory phase were well developed. Radionuclide renal dynamic imaging revealed that the GFR of the transplanted kidney was 36.9 ml/min.

**TABLE 1:** Outcomes of operation.

Item	Outcomes
Operation time	301 min
Blood loss	200 ml
Hot ischemia time	2 min
Cold ischemia time	76 min
Venous anastomosis	23 min
Arterial anastomosis	27 min

## Discussion

Currently, renal autotransplantation (RAT) has been reported as an effective treatment model for managing complex renal/ureteral lesions. The first RAT in humans was performed by Hardy in 1963 to address an extensive ureteral lesion. Over the past 30 years, RAT has been used in the surgical management of complex ureteral lesions, renal artery aneurysms, RVH, renal tumors, low back pain syndrome, and hematuria (Gwon et al., 2017; Ruiz et al., 2017; Doumerc et al., 2018; Haberal et al., 2018; Chen et al., 2020). RVH caused by renal artery stenosis presents as refractory hypertension and with poor drug treatment effect. It generally consists of percutaneous transluminal renal angioplasty (PTRA), *in situ* vascular reconstruction, or renal autotransplantation (RAT). These procedures aim to normalize the blood flow to the affected kidney with the fewest possible surgical complications. However, PTRA may be unsuccessful and even hazardous in patients with dysplastic or atherosclerotic lesions involving infrahilar branches or with inflammatory lesions, such as those associated with TD. RAT has been used as an alternative treatment for the aneurysmal or complex occlusive disease of the renal artery. In the studied case of bilateral renal artery severe stenosis, stenosis of the right renal artery was more severe than that on the left, with the right renal artery accounting for approximately 90% of the stenosis. Narrowing was observed in the proximal renal artery, and because of the poor right kidney function, interventional therapy was difficult. Therefore, we selected RAT as an alternative treatment for this patient to obtain the best long-term results, improve the right kidney blood supply, and obtain compensatory right-side functions. If poor postoperative blood pressure control is achieved, then further treatment of left renal artery stenosis can be administered.

Laparoscopy and robot surgery have been used for donor nephrectomy and renal autotransplantation. Restrictions due to kidney cold preservation *in vivo*, most studies take the kidney extracted from the abdominal cavity and transfer to the bench table, and a separate incision is required to anastomose the renal vessels with the iliac vessels; the kidney is not cold preserved continuously during the operation (Ju et al., 2016; Kumar et al., 2018; Tiong et al., 2018; Ölçücüoğlu, 2020).Currently, some studies reported the use of cold preservation devices for pig intraperitoneal kidney transplantation (Menon et al., 2014; Meier et al., 2018; Samuels et al., 2019). However, they are all in the exploratory stage. In addition, some researchers reported using cold preserve in living donor’s kidney transplants (Territo et al., 2021), but this method requires an additional incision to insert the cold preserve device and donor’s kidney. There were no reports on the use of this device to human completely minimally invasive renal autotransplantation.

In this study, the patient underwent laparoscopic complete intracorporeal renal autotransplantation, the blood pressure was maintained at a normal level, the blood supply of the transplanted kidney recovered to normal levels, and the GFR was significantly recovered. Therefore, we believe that laparoscopic complete intracorporeal renal autotransplantation may be the preferred treatment for severe renal artery stenosis for which interventional therapy is not appropriate.

3D printing technology converts two-dimensional images into specified materials for printing through computer design software (Atalay et al., 2017; Ghazi et al., 2017; Bai et al., 2020). With the concept of biological manufacturing, the application of 3D printing technology in medicine has received increasing attention from researchers worldwide. At present, great progress has been made in reflecting the anatomical details of human organs by 3D printing, which was first used in preoperative planning and surgical simulation of complex operations (Ali et al., 2020; Xu et al., 2020). It has been more commonly used in orthopedics, stomatology, and cranial maxillofacial surgery. Giovinco (Giovinco et al., 2012) applied 3D printing technology in preoperative training for Charcot’s foot orthopedic surgery and achieved good results. In the operation to repair an acetabular fracture, a simulated pelvis model of the patient was printed with 3D technology, and plate pre-bending, screw length measurement, and screw entry direction design were carried out on the model, which greatly reduced the operation time and surgical complications; moreover, the fracture model can be used to train new doctors. 3D printing technology has been applied in urology to print individual 3D solid models according to the shape of the kidney, and the greatest advantage of 3D printing is that artificial implants can be customized accurately and effectively. In this study, we used 3D printing technology to produce a temporary “cold jacket” for the kidney, and its effect was the same as that of *in vitro* cold preservation. This new method of 3D printing technology combined with cold preservation was first successfully applied in the treatment of renal artery stenosis, and this technology has not been previously reported.

PLA, a kind of linear thermoplastic aliphatic polyester, is mainly prepared from starch raw materials through saccharification, fermentation, and certain chemical reactions (Madhavan Nampoothiri et al., 2010; Tyler et al., 2016). It has good thermal stability, solvent resistance, excellent gloss, transparency, resistance to certain bacteria, and flame retardancy (Farah et al., 2016). Because of its unique performance, PLA is widely used in medical tissue engineering research, and it is feasible to make a cold storage device with this material. In this study, our novel cold storage devices overcome the restriction of kidney preservation in the process of complete intracorporeal renal autotransplantation. The kidney was preserved continuously, and the kidney function was protected to the maximum extent; this technology is safe and feasible for use in the process of human autologous kidney transplantation.

However, there are some limitations. First, this study applied 3D printing cold jackets for renal autotransplantation, and the proposed method was only applied to one patient. Additionally, although this new method is very promising, random comparisons with traditional renal autotransplantation were not performed, and the long-term effects and large sample outcomes have not been determined. Finally, whether the technique is suitable for different types of renal transplantation patients requires further clarification; thus, this cold jacket should be optimized in future research.

## Conclusion

A 3D-printed polylactide (PLA) cold jacket for laparoscopic completely intracorporeal renal autotransplantation is a safe, effective, and less complicated method for the treatment of renal artery stenosis; however, the technology is at the exploratory stage and, thus, still has room for improvement. Every step still needs further research and elaboration, the cases need to be carefully selected, and the surgeon must be skilled in laparoscopic techniques, especially laparoscopy vascular sutures.

## Data Availability

The original contributions presented in the study are included in the article/Supplementary Material, further inquiries can be directed to the corresponding authors.

## References

[B13] Ali S. Sirota E. Ali H. Bezrukov E. Okhunov Z. Bukatov M. (2020). Three-Dimensionally Printed Non-Biological Simulator for Percutaneous Nephrolithotomy Training. Scand J. Urol. 54, 349–354. 10.1080/21681805.2020.1773529 32496922

[B1] Agrawal H. Moodie D. Qureshi A. M. Acosta A. A. Hernandez J. A. Braun M. C. (2018). Interventions in Children with Renovascular Hypertension: A 27-year Retrospective Single-center Experience. Congenit. Heart Dis. 13, 349–356. 10.1111/chd.12608 29635838

[B2] Atalay H. A. Canat H. L. Ülker V. Alkan İ. Özkuvanci Ü. Altunrende F. (2017). Impact of Personalized Three-Dimensional (3D) Printed Pelvicalyceal System Models on Patient Information in Percutaneous Nephrolithotripsy Surgery: a Pilot Study. Int. Braz. J. Urol. 43, 470–475. 10.1590/s1677-5538.ibju.2016.0441 28338309 PMC5462137

[B3] Bacic J. Liu T. Thompson R. H. Boorjian S. A. Leibovich B. C. Golijanin D. (2020). Emulating Target Clinical Trials of Radical Nephrectomy with or without Lymph Node Dissection for Renal Cell Carcinoma. Urology 140, 98–106. 10.1016/j.urology.2020.01.039 32142726 PMC7255934

[B4] Bai F. Wu H. Zhang N. Chen J. Wen J. (2020). The Feasibility, Safety, and Efficacy of the Preemptive Indwelling of Double-J Stents in Percutaneous Nephrolithotomy Surgery: A Randomized Controlled Trial. Urol. J. 17, 232–236. 10.22037/uj.v0i0.4957 32309876

[B5] Breda A. Diana P. Territo A. Gallioli A. Piana A. Gaya J. M. (2021). Intracorporeal versus Extracorporeal Robot-Assisted Kidney Autotransplantation: Experience of the ERUS RAKT Working Group. Eur. Urol. 12, S0302–S2838. 10.1016/j.eururo.2021.07.023 34393012

[B6] Buck D. B. Curran T. McCallum J. C. Darling J. Mamtani R. van Herwaarden J. A. (2016). Management and Outcomes of Isolated Renal Artery Aneurysms in the Endovascular Era. J. Vasc. Surg. 63, 77–81. 10.1016/j.jvs.2015.07.094 26386509 PMC4698072

[B7] Cantrell L. A. Oberholzer J. (2018). Robotic Pancreas Transplantation: the State of the Art. Curr. Opin. Organ. Transpl. 23, 423–427. 10.1097/MOT.0000000000000555 29979265

[B8] Chavent B. Duprey A. Lavocat M.-P. Fichtner C. Beraud A.-M. Albertini J.-N. (2017). Renovascular Hypertension: Results in Adulthood of Renal Autotransplantation Performed in Children. Pediatr. Nephrol. 32, 1935–1940. 10.1007/s00467-017-3664-x 28429121

[B9] Chen X. Zhao J. Yuan D. Yang Y. Huang B. (2020). Restenosis of Bilateral Aorta-Renal Saphenous Vein Grafts after the Surgical Repair of Takayasu Arteritis-Induced Bilateral Renal Arteries Stenosis: Case Report. Ann. Vasc. Surg. 62, e1–498. 10.1016/j.avsg.2019.06.021 31449935

[B10] Choi C. I. Kim D. I. Baek S. H. Chung Y. S. Kim D. H. Jeon T. Y. (2018). Initial Experience with Hand-Assisted Laparoscopic Living Donor Nephrectomy: Training and Clinical Practice as a General Surgeon. Transplant. Proc. 50, 3113–3120. 10.1016/j.transproceed.2018.08.052 30577176

[B11] Doumerc N. Beauval J.-B. Roumiguié M. Roulette P. Laclergerie F. Sallusto F. (2018). Total Intracorporeal Robotic Renal Auto-Transplantation: A New Minimally Invasive Approach to Preserve the Kidney after Major Ureteral Injuries. Int. J. Surg. Case Rep. 49, 176–179. 10.1016/j.ijscr.2018.06.017 30015216 PMC6070672

[B12] Farah S. Anderson D. G. Langer R. (2016). Physical and Mechanical Properties of PLA, and Their Functions in Widespread Applications - A Comprehensive Review. Adv. Drug Deliv. Rev. 107, 367–392. 10.1016/j.addr.2016.06.012 27356150

[B14] Ghazi A. Campbell T. Melnyk R. Feng C. Andrusco A. Stone J. (2017). Validation of a Full-Immersion Simulation Platform for Percutaneous Nephrolithotomy Using Three-Dimensional Printing Technology. J. Endourol. 31, 1314–1320. 10.1089/end.2017.0366 29048214

[B15] Giacomoni A. Di Sandro S. Lauterio A. Concone G. Buscemi V. Rossetti O. (2016). Robotic Nephrectomy for Living Donation: Surgical Technique and Literature Systematic Review. Am. J. Surg. 211, 1135–1142. 10.1016/j.amjsurg.2015.08.019 26499052

[B16] Giovinco N. A. Dunn S. P. Dowling L. Smith C. Trowell L. Ruch J. A. (2012). A Novel Combination of Printed 3-dimensional Anatomic Templates and Computer-Assisted Surgical Simulation for Virtual Preoperative Planning in Charcot Foot Reconstruction. J. Foot Ankle Surg. 51, 387–393. 10.1053/j.jfas.2012.01.014 22366474

[B17] Gwon J. G. Kim Y. H. Han D. J. (2017). Real Renal Function after Renal Autotransplantation through the Analysis of Solitary Kidney Autotransplantation Cases. Transplant. Proc. 49, 2055–2059. 10.1016/j.transproceed.2017.09.030 29149960

[B18] Haberal H. B. Tonyali S. Peynircioğlu B. Arici M. Demircin M. Aki F. T. (2018). Renal Autotransplantation with Autologous Saphenous Vein Graft in a Patient with Takayasu Arteritis and Existing Renal Artery Stent in Her Solitary Kidney. Urol. Int. 100, 181–184. 10.1159/000475509 28486233

[B19] Ju X. Li P. Shao P. Lv Q. Wang Z. Qin C. (2016). Retroperitoneal Laparoscopic Nephrectomy Combined with Bench Surgery and Autotransplantation for Renal Cell Carcinoma in the Solitary Kidney or Tumor Involving Bilateral Kidneys: Experience at a Single Center and Technical Considerations. Urol. Int. 97, 473–479. 10.1159/000448594 27732979

[B20] Kari J. A. Roebuck D. J. McLaren C. A. Davis M. Dillon M. J. Hamilton G. (2015). Angioplasty for Renovascular Hypertension in 78 Children. Arch. Dis. Child. 100, 474–478. 10.1136/archdischild-2013-305886 25527520

[B21] Kaths J. M. Echeverri J. Linares I. Cen J. Y. Ganesh S. Hamar M. (2017). Normothermic *Ex Vivo* Kidney Perfusion Following Static Cold Storage-Brief, Intermediate, or Prolonged Perfusion for Optimal Renal Graft Reconditioning? Am. J. Transpl. 17, 2580–2590. 10.1111/ajt.14294 28375588

[B22] Kim M. J. Lee K. W. Park J. B. Kim S. J. (2017). Hand-Assisted Laparoscopic Nephrectomy and Auto-Transplantation for a Hilar Renal Artery Aneurysm: A Case Report. VSI 33, 84–87. 10.5758/vsi.2017.33.2.84 28691001 PMC5493192

[B23] Kishore T. A. Kuriakose M. J. Pathrose G. Raveendran V. Kumar K. V. Unni V. N. (2020). Robotic Assisted Kidney Transplantation in Grafts with Multiple Vessels: Single center Experience. Int. Urol. Nephrol. 52, 247–252. 10.1007/s11255-019-02305-z 31586280

[B24] Kubota R. Araki M. Wada K. Kawamura K. Maruyama Y. Mitsui Y. (2020). Robotic Renal Autotransplantation: A Feasibility Study in a Porcine Model. Acta Med. Okayama 74, 53–58. 10.18926/AMO/57953 32099249

[B25] Kumar A. Chaturvedi S. Gulia A. Maheshwari R. Dassi V. Desai P. (2018). Laparoscopic Live Donor Nephrectomy: Comparison of Outcomes Right versus Left. Transplant. Proc. 50, 2327–2332. 10.1016/j.transproceed.2018.03.034 30316352

[B26] Lin D. Xiang T. Qiu Q. Leung J. Xu J. Zhou W. (2020). Aldehyde Dehydrogenase 2 Regulates Autophagy via the Akt-mTOR Pathway to Mitigate Renal Ischemia-Reperfusion Injury in Hypothermic Machine Perfusion. Life Sci. 253, 117705. 10.1016/j.lfs.2020.117705 32334008

[B27] Madhavan Nampoothiri K. Nair N. R. John R. P. (2010). An Overview of the Recent Developments in Polylactide (PLA) Research. Bioresour. Techn. 101, 8493–8501. 10.1016/j.biortech.2010.05.092 20630747

[B28] Meier R. P. H. Piller V. Hagen M. E. Joliat C. Buchs J.-B. Nastasi A. (2018). Intra-Abdominal Cooling System Limits Ischemia-Reperfusion Injury during Robot-Assisted Renal Transplantation. Am. J. Transpl. 18, 53–62. 10.1111/ajt.14399 PMC5763420 28637093

[B29] Menon M. Sood A. Bhandari M. Kher V. Ghosh P. Abaza R. (2014). Robotic Kidney Transplantation with Regional Hypothermia: A Step-by-step Description of the Vattikuti Urology Institute-Medanta Technique (IDEAL Phase 2a). Eur. Urol. 65, 991–1000. 10.1016/j.eururo.2013.12.006 24388099

[B30] Ölçücüoğlu E. (2020). Comparing the Complications of Laparoscopically Performed Simple, Radical and Donor Nephrectomy. Turk J. Med. Sci. 50, 922–929. 10.3906/sag-1910-120 32490652 PMC7379416

[B31] Perkins S. Q. Giffen Z. C. Buck B. J. Ortiz J. Sindhwani P. Ekwenna O. (2018). Initial Experience with the Use of a Robotic Stapler for Robot-Assisted Donor Nephrectomy. J. Endourol. 32, 1054–1057. 10.1089/end.2018.0461 30160167

[B32] Pomy B. Glousman B. Macsata R. (2020). Management of Bilateral Renal Artery Aneurysms with Laparoscopic Nephrectomy, *Ex Vivo* Reconstruction, and Autotransplantation in a Woman Planning Pregnancy. J. Vasc. Surg. Cases, Innov. Tech. 6, 126–128. 10.1016/j.jvscit.2020.01.006 32123779 PMC7037528

[B33] Raborn J. McCafferty B. J. Gunn A. J. Moawad S. Mahmoud K. Aal A. K. A. (2020). Endovascular Management of Neurofibromatosis Type I-Associated Vasculopathy: A Case Series and Brief Review of the Literature. Vasc. Endovascular Surg. 54, 182–190. 10.1177/1538574419885257 31672102

[B34] Ruiz M. Hevia V. Fabuel J.-J. Fernández A.-A. Gómez V. Burgos F.-J. (2017). Kidney Autotransplantation: Long-Term Outcomes and Complications. Experience in a Tertiary Hospital and Literature Review. Int. Urol. Nephrol. 49, 1929–1935. 10.1007/s11255-017-1680-1 28828690

[B35] Safdar O. Alaifan F. Alshammakh S. Hakami M. Alghaithi D. F. (2020). Diagnostically Challenging Case of Renal Artery Stenosis in a Pediatric Patient. Cureus 12, e6538. 10.7759/cureus.6538 31929955 PMC6939964

[B36] Samuels J. A. Zavala A. S. Kinney J. M. Bell C. S. (2019). Hypertension in Children and Adolescents. Adv. Chronic Kidney Dis. 26, 146–150. 10.1053/j.ackd.2019.02.003 31023449

[B37] Seitz C. Hiess M. (2016). Robot-assisted Renal Surgery: Current Status and Future Directions. Robot Surg. 3, 1–12. 10.2147/RSRR.S71328 30697551 PMC6193442

[B38] Siena G. Vignolini G. Mari A. Li Marzi V. Caroassai S. Giancane S. (2019). Full Robot-Assisted Living Donor Nephrectomy and Kidney Transplantation in a Twin Dedicated Operating Room: Initial Experience from a High-Volume Robotic Center. Surg. Innov. 26, 449–455. 10.1177/1553350619835429 31018770

[B39] Smith M. Lazar A. Morrissey N. Ratner L. (2020). Laparoscopic Nephrectomy with *Ex Vivo* Repair of Aneurysm and Autotransplantation. J. Vasc. Surg. Cases, Innov. Tech. 6, 24–26. 10.1016/j.jvscit.2019.11.009 32055758 PMC7005476

[B40] Spinoit A.-F. Moreels N. Raes A. Prytula A. De Groote R. Ploumidis A. (2019). Single-setting Robot-Assisted Kidney Transplantation Consecutive to Single-Port Laparoscopic Nephrectomy in a Child and Robot-Assisted Living-Related Donor Nephrectomy: Initial Ghent Experience. J. Pediatr. Urol. 15, 578–579. 10.1016/j.jpurol.2019.08.005 31519482

[B41] Territo A. Piana A. Fontana M. Diana P. Gallioli A. Gaya J. M. (2021). Step-by-step Development of a Cold Ischemia Device for Open and Robotic-Assisted Renal Transplantation. Eur. Urol. 21, 01795–1804. 10.1016/j.eururo.2021.05.026 34059396

[B42] Tiong H. Y. Goh B. Y. S. Chiong E. Tan L. G. L. Vathsala A. (2018). Robotic Kidney Autotransplantation in a Porcine Model: a Procedure-specific Training Platform for the Simulation of Robotic Intracorporeal Vascular Anastomosis. J. Robotic Surg. 12, 693–698. 10.1007/s11701-018-0806-5 29605864

[B43] Tyler B. Gullotti D. Mangraviti A. Utsuki T. Brem H. (2016). Polylactic Acid (PLA) Controlled Delivery Carriers for Biomedical Applications. Adv. Drug Deliv. Rev. 107, 163–175. 10.1016/j.addr.2016.06.018 27426411

[B44] Urbanellis P. Hamar M. Kaths J. M. Kollmann D. Linares I. Mazilescu L. (2020). Normothermic *Ex Vivo* Kidney Perfusion Improves Early DCD Graft Function Compared with Hypothermic Machine Perfusion and Static Cold Storage. Transplantation 104, 947–955. 10.1097/TP.0000000000003066 31815900

[B45] Xu Y. Yuan Y. Cai Y. Li X. Wan S. Xu G. (2020). Use 3D Printing Technology to Enhance Stone Free Rate in Single Tract Percutaneous Nephrolithotomy for the Treatment of Staghorn Stones. Urolithiasis 48, 509–516. 10.1007/s00240-019-01164-8 31616985

